# Immunopeptidomics in the Era of Single-Cell Proteomics

**DOI:** 10.3390/biology12121514

**Published:** 2023-12-12

**Authors:** Rupert L. Mayer, Karl Mechtler

**Affiliations:** 1 Research Institute of Molecular Pathology (IMP), Vienna BioCenter, 1030 Vienna, Austria; 2 Gregor Mendel Institute of Molecular Plant Biology (GMI), Austrian Academy of Sciences, Vienna BioCenter (VBC), 1030 Vienna, Austria; 3 Institute of Molecular Biotechnology (IMBA), Austrian Academy of Sciences, Vienna BioCenter (VBC), 1030 Vienna, Austria

**Keywords:** antigen discovery, immunopeptidomics, single-cell proteomics, mass spectrometry, vaccine development, MHC, HLA, liquid chromatography, data-independent acquisition

## Abstract

**Simple Summary:**

Fragments of pathogens, such as viruses and bacteria as well as cancer cells, are presented to the immune system via the major histocompatibility complex (MHC). Immunopeptidomics is a mass spectrometry-based technique that allows us to identify which peptide fragments of pathogens or aberrant mutated proteins are presented and potentially recognized by the immune system. Recent technological developments in mass spectrometry as well as sample preparation, peptide separation, and data analysis have substantially propelled the field of immunopeptidomics further and often initially were developed or incentivized for the measurement of single-cell proteomics. This perspective describes how developments in single-cell proteomics have benefitted immunopeptidomics and how further implementations could still boost sensitivity, and it will explore future directions and trends in immunopeptidomics.

**Abstract:**

Immunopeptidomics, as the analysis of antigen peptides being presented to the immune system via major histocompatibility complexes (MHC), is being seen as an imperative tool for identifying epitopes for vaccine development to treat cancer and viral and bacterial infections as well as parasites. The field has made tremendous strides over the last 25 years but currently still faces challenges in sensitivity and throughput for widespread applications in personalized medicine and large vaccine development studies. Cutting-edge technological advancements in sample preparation, liquid chromatography as well as mass spectrometry, and data analysis, however, are currently transforming the field. This perspective showcases how the advent of single-cell proteomics has accelerated this transformation of immunopeptidomics in recent years and will pave the way for even more sensitive and higher-throughput immunopeptidomics analyses.

## 1. Introduction

Immunopeptidomics as the study of MHC-presented peptides has been around for decades, starting with the first mass spectrometry-based description of immunopeptides by Hunt and colleagues in 1992 [1]. Mass spectrometry allows the untargeted and high-throughput investigation of the immunopeptidome to comprehensively describe the epitope pool that the immune system encounters and can react to. Hence, this technique presents an invaluable tool for the development of immune-based therapeutics, such as vaccines, CAR T-cell therapies, and others [2,3,4,5] and has contributed substantially to global epitope knowledge over the last few decades [6]. The measurement of MHC-presented peptides is currently considered a highly impactful method within the recent surge of interest in vaccine development due to the SARS-CoV-2 pandemic [7,8] and to increasing development of antimicrobial resistance (AMR) [9,10,11], as well as the further emerging interest in effective and well-tolerated cancer immune therapy [12,13]. Cancer immunotherapies often aim to target so called neoantigens, which arise due to somatic mutations in the tumor that are distinct from self and could be recognized by the immune system as non-self to trigger tumor cell degradation.

In the last 5–10 years, the field has seen unprecedented growth and development, which can be in large part explained by quantum leaps in mass spectrometry instrumentation and data analysis as well as improvements in sample preparation and liquid chromatography. When a few scientists were entertaining the idea of proteomic measurements of single individual cells, the vast majority of the field deemed this endeavor impossible. Today, we know that indeed it is very much possible albeit still challenging to do single-cell proteomics (SCP) [14,15,16,17,18,19,20,21,22,23].

While only very large cells such as oocytes could be analyzed in the early days of SCP [24], smaller cells such as mouse embryonic stem cells can also be successfully analyzed today at a satisfactory analytical depth to showcase cellular heterogeneity [25]. Immunopeptidomics faces similar challenges as SCP as the quantity of MHC-presented peptides is typically very low, and in the past, hundreds of millions of cells per individual sample were required to achieve a somewhat comprehensive picture of MHC-presented peptides in a sample. Reducing the number of cells or amount of tissue required for immunopeptidomic analysis is highly desirable, as it would allow us to analyze needle biopsies or reduce growth time and the required volume of cell culture medium for cellular experiments. This has also implications for other proteomics subdomains, such as the field of cross-linking mass spectrometry, which would also substantially gain from improved sensitivity in order to characterize molecular interactions in cellular subpopulations under endogenous conditions or potentially even for single cells one day.

In this perspective, it is presented how technological advancements initially applied to SCP have aided the maturation and development of immunopeptidomics; it is highlighted how further implementations of current practices in SCP could also benefit immunopeptidomics, and future potential directions in the field of immunopeptidomics are explored.

## 2. Miniaturization and Acceleration of Immunopeptidomics Sample Preparation

In order to avoid protein and peptide sample losses, surface exposure of the protein or peptide sample needs to be minimized. This is true in general for bulk proteomics but put to the extreme in SCP where only a single additional sample transfer step by pipetting will reduce protein identifications by up to 50% [15]. Different approaches have been implemented in single-cell proteomics to reduce surface-related peptide losses, including (i) sample volume reduction to reduce surface area in contact with the sample container, (ii) use of mass spectrometry-compatible detergents to retain peptides in solution [26,27], or (iii) one-pot sample preparation to minimize surface contact [15]. Both classical bulk proteomics and immunopeptidomics samples were previously typically prepared in 0.5–15 mL tubes in at least several hundreds of µLs of final volume. In single-cell proteomics these volumes have been substantially downscaled to 1 µL or below, which drastically reduces surface exposure, and typically inert materials such as glass or Teflon are chosen for sample containers. In contrast to single-cell proteomics, for which sample surface reduction is rather straightforward, immunopeptidomics requires specific immunoprecipitation (IP) of the intact MHC–peptide complex before acidic release of the immunopeptide from the MHC complex and typically further purification of these peptides from the MHC alpha chain and the beta-2 microglobulin molecules. Immunopeptidomics samples are hence exposed to more surfaces than bulk or single-cell samples, and minimizing the exposed surface area becomes even more important.

While not strictly limited to SCP, the use of 96- and 384-well plates is very common these days in SCP [15,16,25,28] and was first implemented in immunopeptidomics by Chong et al. in 2018 [29]. Their platform consisted of stacked 96-well filter plates that contained MHC–peptide-specific antibody beads over which cell and tissue lysates were passed to pull down the intact MHC–peptide complexes. MHC–peptide pull-down was followed by washing, acidic complex dissociation, and C18 purification of the MHC-presented peptides before LC–MS/MS analysis. In contrast to earlier studies, for which typically hundreds of millions of cells were used, they explored the analysis of as little as 10 million cells on their miniaturized platform and obtained up to 1846 MHC class I- and 2633 MHC class II-presented peptides for CD165 cells [29]. More recently published low-input immunopeptidomics platforms also use a well plate-based sample preparation strategy [30,31]. Another advantage of the well plate format besides increased sensitivity is the improved throughput, since up to 96 samples can be processed in parallel, which reduces preparation times per sample drastically. Without the need to enzymatically digest intact proteins, sample preparation can typically be sped up to be completed within 1–2 days. The group of Pouya Faridi recently described an elegant approach termed SAPrIm, which presents a mid-throughput platform. This platform utilizes a semi-automated platform harnessing a KingFisher robot to isolate immunopeptidomes via anti-HLA antibodies attached to highly porous magnetic microbeads and the data-independent acquisition (DIA) method with Spectronaut (SN) as the data analysis platform [32]. Similar to chip-based approaches in SCP and low-input proteomics [23,33,34,35], the group of Bassani-Sternberg very recently developed a chip-based system for immunopeptidomics sample preparation. This system uses micropillar arrays to demonstrate unsurpassed sensitivity in immunopeptidomics with around 4000 MHC class I peptides identified for only 200,000 RA957 cells, while only 2700 peptides were identified in comparison via the column-based method [36]. They could also identify more than 5000 immunopeptides from as little as 5 mg of melanoma tissue. Implementation of MS-compatible detergents such as *n*-dodecyl-beta-maltoside (DDM) has been reported to increase the number of identified protein and peptide species in single-cell proteomics [37], while it has not been reported in immunopeptidomics to date, to the best of the authors’ knowledge. Implementation of DDM could potentially improve analytical depth, particularly for the longer and more hydrophobic MHC class II-presented immunopeptides, when applied upon, and after acidic elution of, MHC-bound peptides.

## 3. Improving Peptide Separation and Ionization for Increased Sensitivity

Following highly efficient sample preparation, peptide separation prior to mass spectrometric detection is another critical step in both SCP and immunopeptidomics workflows. In single-cell proteomics, current approaches utilize ultra-low-flow liquid chromatography with flow rates during peptide elution of 100 nL/min or below [38,39,40,41]. This reduced elution volume boosts peptide concentrations, thereby increasing peptide ionization efficiencies and consequently signal intensities within the mass spectrometer. These ultra-low flows, however, present the challenge of low throughput when static flow rates are employed. Different approaches are being utilized in SCP to overcome this reduced throughput, including (i) higher flow rates before and after active peptide elution [40], (ii) the use of one or more trapping columns [42], and (iii) the use of multiple trapping and analytical columns [39]. 

One study from the Kelly lab achieved a throughput of 200 samples per day with a dual-column system utilizing two trapping and analytical columns, while employing only a single UltiMate 3000 RSLCnano pump module, including loading and nano pump at a flow rate of only 80 nL/min [39]. This system also included a sophisticated fluidic and switching setup, enabling the measurement of up to 200 samples/day at run-to-run times of 7 min or higher proteomic coverage for longer analysis times. Another approach co-developed by Thermo Fisher (Germering, Germany) and our lab with lead authors Zheng and Matzinger utilizes a standard LC setup including a Vanquish Neo LC in combination with a 15 cm long analytical column with a 50 μm inner diameter (i.d.) and a 5 mm trapping cartridge, which are all commercially available [40]. Using high flow rates for loading on the trapping column of 200 µL/min and 500 nL/min on the analytical column during column equilibration and washing facilitates run-to-run times of 14.4 min and therefore 100 samples/day at a proteomic depth of up to 1700 protein groups for HeLa single cells. The nanoflow dual-trap single-column (nanoDTSC) platform developed by Kreimer and colleagues utilizes, as indicated by the name, two trapping columns and a single 15 cm (length) × 75 µm (i.d.) analytical PepSep column [42]. The platform features the widely available UltiMate 3000 RSLCnano, which allows easy implementation of the platform with excellent proteomic coverage of up to 2000 protein groups when analyzing multiple single cells using dia-PASEF and DIA-NN.

Considering the choice of the analytical column, several parameters and attributes have to be accounted for, including (i) column length and inner diameter and consequently the resulting backpressure for application of high flow rates during equilibration and washing, (ii) packing material, (iii) the price of the column, (iv) the robustness of the column, and (v) suitability for low flow rates. Columns that have been very successfully used in SCP as well as limited input proteomics include the 5 cm Aurora Rapid150, the 15 cm Aurora Elite, and the 25 cm Aurora Ultimate columns from IonOpticks (Fitzroy, Australia), as well as the 5.5 cm High-Throughput [43], the 50 cm Low-Load [25,44], and the 50 cm µPAC Neo columns [43] from Thermo Fisher Scientific (Waltham, MA, USA). A more conventional column we utilized successfully for single-cell proteomics is the 15 cm (length) × 50 µm (i.d.) Acclaim PepMap 100 column from Thermo Fisher Scientific [40]. All these columns present excellent options for highly efficient separation of highly limited peptide samples with better separation power for the longer columns (Aurora 15 & 25 cm, µPAC Neo 50 cm) and higher throughput for the shorter columns (Aurora Rapid150 and High-Throughput µPAC). Most immunopeptidomics studies utilized self-packed or well-established chromatographic columns, which could present an opportunity to further improve sensitivity for immunopeptidomics analyses, while rigorous specific testing for the mostly non-tryptic immunopeptides will be required. Beyond the use of different chromatographic columns, the use of 0.5% acetic acid over 0.1% formic acid as acidifier has recently also been very successfully explored in bottom-up [45] and single-cell proteomics [46], as peptide and protein identifications (IDs) could slightly be improved as well as precision enhanced. While the non-tryptic nature of immunopeptides could prohibit the gain evidenced in bulk- and single-cell proteomics, it might still represent another tool to improve immunopeptidomics sensitivity.

In order to deliver both ultra-low flow rates and high flows for equilibration and washing, two LC systems have established themselves in the single-cell proteomics field, including the Vanquish Neo from Thermo Fisher Scientific and the Evosep One from Evosep (Odense, Denmark). The Vanquish Neo allows scientists to employ highly dynamic and accurate flow rates from 100 nL/min up to 100 µL/min, allowing setup of the aforementioned ultra-low flow methods with a throughput that is still high. While the Vanquish Neo is a rather conventional LC system that allows the use of trap-and-elute schemes or direct injection onto the analytical column, the Evosep One features single-use tips (Evotips) on which the sample is stored before being eluted onto a storage loop before being transferred to the analytical column during the analytical run. The Evosep features minimal to no carryover between samples due to the elimination of carryover via the trapping column. The Evosep One comes with predetermined gradients that cannot be adjusted by the user but offer a wide variety of different gradients to choose from for different applications, ranging from ultrashort gradients of 3.2 min for analysis of abundant analytes to so-called Whisper methods that employ 100 nL/min flow rates during the active gradient and that are mostly used for SCP or limited input proteomics.

## 4. Evolution of Mass Spectrometers for SCP and Immunopeptidomics

**Figure 1:**
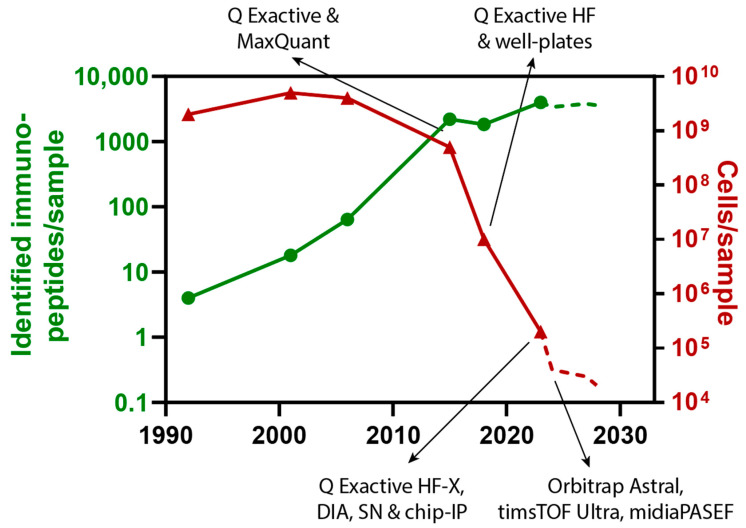
Evolution of sensitivity in MS-based immunopeptidomics. Illustration of the continuous improvement of immunopeptidomics sensitivity over the last 32 years. Green dots indicate the number of identified immunopeptides/sample, while red triangles illustrate the required number of cells/sample. Data points from 2010 onwards are assigned with implemented technological advancements. The dashed lines represent a projection of the authors of how the sensitivity in MS-based immunopeptidomics could improve through new technologies, such as the recently introduced Orbitrap Astral and timsTOF Ultra mass spectrometers as well as the novel midiaPASEF acquisition method as developed in the Tenzer lab together with Bruker. Datapoints are derived from the following studies (from left to right): [1,29,36,49,63,64].

The greatest impact on the evolution of sensitivity in proteomics in general, and immunopeptidomics and SCP in particular, has undoubtedly come from the development of new mass spectrometers. Particularly the introduction of the first commercial Orbitrap instruments in 2005 [47] and the workhorse Q Exactive in 2011 [48] by Thermo Scientific have laid the foundation for modern-day sensitive proteomics applications. As indicated in Figure 1, the number of immunopeptides/sample increased roughly by two orders of magnitude after the Q Exactive orbitrap mass spectrometer and MaxQuant as powerful, reliable, and sensitive tools became available for immunopeptidomics [49]. The MaxQuant software package (https://www.maxquant.org/, accessed on 5 December 2023) was developed by Jürgen Cox and Matthias Mann and published in 2008, representing an accurate and reliable as well as user-friendly and freely available software solution for searching and analyzing high-resolution mass spectrometry data and still remains one of the most predominantly used software packages in the field of proteomics globally [50,51].

Since 2017, however, the predominance of Orbitrap machines in the field of proteomics has been challenged substantially by the introduction of the timsTOF Pro instrument from Bruker (Billerica, MA, USA), which allows the measurement of peptide ion mobilities as a fourth dimension next to *m*/*z*, intensity, and retention time [52]. Additionally, the timsTOF Pro delivered unheard of speed at the time with up to 100 fragmentation scans/s. In 2022, the Matthias Mann lab, together with Bruker, further developed the timsTOF Pro to enhance sensitivity with the aim of enabling single-cell proteomics. The result of these efforts is the timsTOF SCP (Single-Cell Proteomics) instrument, which features substantially improved ion transmission and an additional higher pressure vacuum stage, delivering an over 4× higher ion current and, together with the EvosepOne liquid chromatography system, up to 100× higher sensitivity [16]. A recent work from the Carr lab has utilized the timsTOF SCP to showcase its high sensitivity for immunopeptidomics applications yielding up to 15,000 immunopeptide IDs from as little as 4 × 10^7^ cells with a single-shot LC–MS/MS method [30] when using data-dependent acquisition and searching against a comprehensive database containing roughly 290,000 entries. The authors utilized the availability of ion mobility to specifically target singly charged peptides, next to doubly, triply, and quadruply charged ones, while avoiding the fragmentation of non-peptide contaminants. They further show that the timsTOF SCP yields about twice as many immunopeptide IDs as the Exploris 480 with FAIMS (high-field asymmetric waveform ion mobility spectrometry). They also show that still around 3000 peptides can be identified with their method when using only the equivalent of 1 × 10^6^ cells. As mentioned earlier in the manuscript, the team of Bassani-Sternberg showed that a high number of IDs can be still attained using even less starting material (2 × 10^5^ cells) with a less sensitive instrument, that is, the Q Exactive HF-X in their case [36]. However, they utilized data-independent acquisition (DIA) and recorded libraries including larger cell quantities to circumvent the reduced sensitivity of the instrument. They furthermore utilized a highly efficient chip-IP platform to minimize adsorptive losses during sample preparation. While it is of relevance to demonstrate high numbers of peptide identifications, this only serves as a proxy for the ability of the method to identify low-abundant neoepitopes or cancer-specific epitopes that typically hide deep in the pool of self-epitopes. Both works demonstrate impressively that their respective approach can identify non-canonical and cancer-specific immunopeptides and potentially also neoepitopes. 

Beyond dia-PASEF [53], which is only available on timsTOF instruments and consecutively cycles through predefined frames in the *m*/*z* and ion mobility dimension, several other more-refined DIA-based methods have been presented recently for Bruker instruments, including Slice-PASEF [54] and Synchro-PASEF [55], which utilize a larger fraction of the ion current thereby boosting sensitivity. Another approach termed midiaPASEF utilizes mobility-specific micro-encoding to design overlapping quadrupole windows that optimally cover the entire ion population in the *m*/*z*–ion mobility plane [56]. With these overlapping ion mobility-encoded quadrupole windows, the system can determine the precursor *m*/*z* for every fragment with an error of less than 2 *m*/*z*. The associated MIDIAID pipeline allows us then to do multidimensional deconvolution of the DIA spectra to generate midia-PASEF files exporting highly specific DDA-like MS/MS spectra, which can then be readily utilized for de novo sequencing and are compatible with established software solutions such as PEAKS studio (https://www.bioinfor.com/peaks-studio/, accessed on 5 December 2023), FragPipe (https://fragpipe.nesvilab.org/, accessed on 5 December 2023), or MASCOT (https://www.matrixscience.com/, accessed on 5 December 2023), showing exciting potential for many applications, including phosphopeptide analysis and immunopeptidomics.

This midia-PASEF technology requires very fast switching of the quadrupole and is therefore not available on older timsTOF instruments. The newly released timsTOF Ultra from Bruker, however, is equipped with the necessary VistaScan acquisition capability and will allow utilizing midia-PASEF once fully launched. Besides the option to run midia-PASEF, the timsTOF Ultra features even higher sensitivity than its predecessor the timsTOF SCP and faster scan speed, leading to a maximum of 300 Hz for MS/MS. The timsTOF Ultra is therefore even better suited for single-cell proteomics and is also highly expected to be a gamechanger in the immunopeptidomics field. Another milestone mass spectrometer released very recently by Thermo Fisher Scientific is the Orbitrap Astral. In contrast to earlier Orbitrap instruments, this machine features an additional novel mass analyzer termed the Asymmetric Track Lossless (Astral) analyzer next to the Orbitrap. While the Orbitrap provides ultra-high resolution, the Astral analyzer provides MS/MS acquisition rates of up to 200 Hz at high sensitivity and still-sufficient resolution to resolve the reporter ions observed during the analysis of TMT-based projects (tandem mass tags). These tandem mass tags function as sample barcodes that allow us to combine and simultaneously measure up to 18 samples in a single LC–MS/MS run [57,58]. The labels applied to the different samples all lead to the same increase in mass initially and can only be distinguished by small mass differences in reporter ions upon peptide fragmentation resulting in distinct quantitative signals for each sample. Therefore, sample complexity before fragmentation remains unchanged, leading to a boost in peptide signal intensities due to stacking of analyte signals. This effect has been heavily utilized in the field of SCP as spearheaded by Budnik and colleagues [20,59,60] and has also found use in several immunopeptidomics works [2,61]. It is therefore highly beneficial that also the new Astral analyzer facilitates the use of TMT. The Astral’s high sensitivity and speed make this impressive instrument ideally suited again for SCP and immunopeptidomics. A recent publication by the manufacturers demonstrates the identification of ca. 5700 protein groups from as little as 250 pg HeLa digest using DIA and a library to search against [44], which represents the highest number of protein groups reported to date for 250 pg HeLa. Also, excellent relative quantification is reported for a triple proteome mix of HeLa, yeast, and *E. coli* digest. An additional study on the Astral from the Olsen lab demonstrates the identification of around 5200 proteins from single HeLa cells, which is again the highest number of protein IDs for a single HeLa cell that the authors are aware of [62].

## 5. Discussion

The field of immunopeptidomics and single-cell proteomics are currently arguably the two most dynamic and intensely discussed subfields within the proteomics world. Many inventions initially developed to increase sensitivity to facilitate single-cell analysis have also been applied to the field of immunopeptidomics such as the use of the timsTOF SCP, microchip-based sample preparation, and other innovations. Similar to SCP, the sensitivity gain in immunopeptidomics over the last decades is nothing short of remarkable and has led to implementation of this technique into vaccine design pipelines or the development of cancer immunotherapies. While already a highly potent methodology, immunopeptidomics still has ample potential for even improved sensitivity when all available tools are combined, such as chip-based IP, ultra-low-flow LC, and cutting-edge mass spectrometers such as the timsTOF Ultra or the Orbitrap Astral. Since no peer-reviewed manuscript on the latter has been published until this perspective was written, direct comparison between these two new flagship instruments from Thermo Fisher Scientific and Bruker is not possible. Preliminary results in the lab of the authors of this perspective indicate, however, very comparable sensitivity of the timsTOF Ultra and the Orbitrap Astral. It will be very enticing to see which level of performance both instruments can reach in practice in different labs around the world for both single-cell proteomics and immunopeptidomics. In light of all these promising tools available for immunopeptidomics, the authors expect that it will be possible in the near future to identify thousands of immunopeptides from as little as 20,000 cells, which is ten times less than the current most sensitive approach. It would allow us to obtain comprehensive imunopeptidomes from even smaller needle biopsies than current techniques already allow and would also increase comprehensiveness of regular needle biopsies to uncover clinically relevant neoantigens, cancer-associated antigens, and bacterial or viral antigens. The analysis of smaller biopsies could also pave the way for spatial immunopeptidomics, for which clinicians could sample a tumor in different locations to obtain information about the differential presentation of MHC ligands in different tumor areas.

## 6. Conclusions

The authors conclude that immunopeptidomics, while already a very refined discipline in the greater field of proteomics, still has tremendous potential to further improve throughput and sensitivity, not least due to lessons that can be learned from SCP: either small adaptations, such as changes in acidifiers or the use of MS-compatible detergents, or quantum leaps, such as the use of new mass spectrometers. The future for immunopeptidomics is surely bright and its clinical potential in high demand for mankind in the era of pandemics and failing antibiotics.
